# Surgical treatment of localized gingival recessions using coronally advanced flaps with or without subepithelial connective tissue graft

**DOI:** 10.4317/medoral.21043

**Published:** 2015-11-22

**Authors:** Ricardo Bellver-Fernández, Ana-María Martínez-Rodriguez, Claudio Gioia-Palavecino, Raul-Guillermo Caffesse, Miguel Peñarrocha

**Affiliations:** 1 DDS, Master in Oral Surgery and Implantology, CEU Cardenal-Herrera University, Valencia, Spain; 2 DDS, PhD, University of Murcia, Murcia, Spain; 3 DDS, Master in Integrated Odontology and Implantology, University of Murcia, Murcia, Spain; 4 Visiting Professor, Postgraduate program in Periodontics, Faculty of Dentistry, Madrid Complutense University, Madrid, Spain; 5 Chairman of Oral Surgery, Department of Stomatology, Faculty of Dentistry, University of Valencia, Valencia, Spain

## Abstract

**Background:**

A coronally advanced flap with subepithelial connective tissue graft is the gold standard surgical treatment of gingival recessions, since it offers a higher probability of achieving complete root coverage compared with other techniques. However, optimum short- and middle-term clinical results have also been obtained with coronally advanced flaps alone. The aim of the present study was to evaluate the results obtained by the surgical treatment of localized gingival recessions using coronally advanced flaps with or without subepithelial connective tissue graft.

**Material and Methods:**

The reduction of recession height was assessed, together with the gain in gingival attachment apical to the recession, and total reduction of recession, in a comparative study of two techniques. Twenty-two gingival recessions were operated upon: 13 in the control group (coronally advanced flap) and 9 in the test group (coronally advanced flap associated to subepithelial connective tissue graft).

**Results:**

After 18 months, the mean reduction of recession height was 2.2 ± 0.8 mm in the control group and 2.3 ± 0.7 mm in the test group, with a mean gain in gingival attachment of 1.3 ± 0.9 mm and 2.3 ± 1.3 mm, respectively. In percentage terms, the mean reduction of recession height was 84.6 ± 19.6% in the control group and 81.7 ± 17.8% in the test group, with a mean gain in gingival attachment of 20.5 ± 37.4% and 184.4 ± 135.5%, respectively.

**Conclusions:**

Significant reduction of gingival recession was achieved with both techniques, though the mean gain in gingival attachment (in mm and as a %) was greater in test group.

**Key words:**Gingival recession, coronally advanced flap, subepthelial connective tissue graft.

## Introduction

Gingival recession is displacement of the gingival marginal apical to the cementoenamel junction (CEJ), with exposure of the root surface of the tooth (1). Such recession is frequent both in populations with good oral hygiene and in populations with deficient oral hygiene (2,3), and is conditioned by a number of triggering and predisposing factors - the most frequent of which are aggressive tooth brushing and malpositioned teeth (4,5). The treatment of gingival recession is decided mainly for aesthetic reasons, but also because of dentin hypersensitivity problems. The demand for treatment is moreover increasing (6).

A number of surgical techniques have been described and used for root covering: lateral sliding flap (7), double papilla positioned flap (8), free gingival graft (9), lateral positioned flap (10), coronally advanced flap with free gingival graft (11), coronally advanced flap with subepithelial connective tissue graft (12), semi lunar flap (13), and coronally positioned flap (14). A coronally advanced flap (CAF) with subepithelial connective tissue graft (SCTG) is the gold standard, since it offers a greater probability of achieving complete root coverage compared with other techniques (15). However, optimum short- and middle-term clinical results have also been obtained with only a coronally advanced flap (14,15).

The present study was carried out to determine whether the surgical treatment of localized gingival recession using coronally advanced flaps with or without subepithelial connective tissue graft is able to improve the clinical results in terms of decreased gingival recession, gain in keratinized gingiva, and total reduction of recession.

## Material and Methods

- Study design

A retrospective, controlled clinical study was made to compare the results obtained with two surgical techniques. The inclusion criteria were: a) the use of one same coronally advanced flap design with or without subepithelial connective tissue grafting; b) a minimum follow-up of 18 months; c) single tooth recession; d) Miller class I and II recession and Miller class III recession with papillary retraction < 2 mm; and e) a complete case history and surgical report.

The study initially comprised 87 patients with gingival recession subjected to surgical treatment between May 2009 and December 2012 in the Claudio Gioia Clinic (Elche, Spain). Of these, 70 were discarded for the following reasons: coronally advanced flap procedures involving two or more adjacent recessions (22 patients); less than 18 months of follow-up (12 patients); evaluation or reevaluation not made by the same examiner (10 patients); patients subjected to non-autologous subepithelial grafting (14 patients); and patients treated with modified versions of the coronally advanced flap technique (12 patients).

 The control group (CG) underwent coronally advanced flap (CAF) treatment, while the test group (TG) underwent coronally advanced flap surgery with subepithelial connective tissue graft (CAF+SCTG).

The study was approved by the Ethics Committee of the University of Valencia (Valencia, Spain) (procedure number: H1404212828195), and written informed consent was obtained from the participants.

- Clinical parameters 

The following data were recorded for each patient: age (at the time of surgery), gender, smoking habit (nonsmoker or smoker of over 10 cigarettes/day), periodontal disease (presence or absence of controlled periodontal disease before the operation), and recession tooth ( Table 1).

**Table 1:**
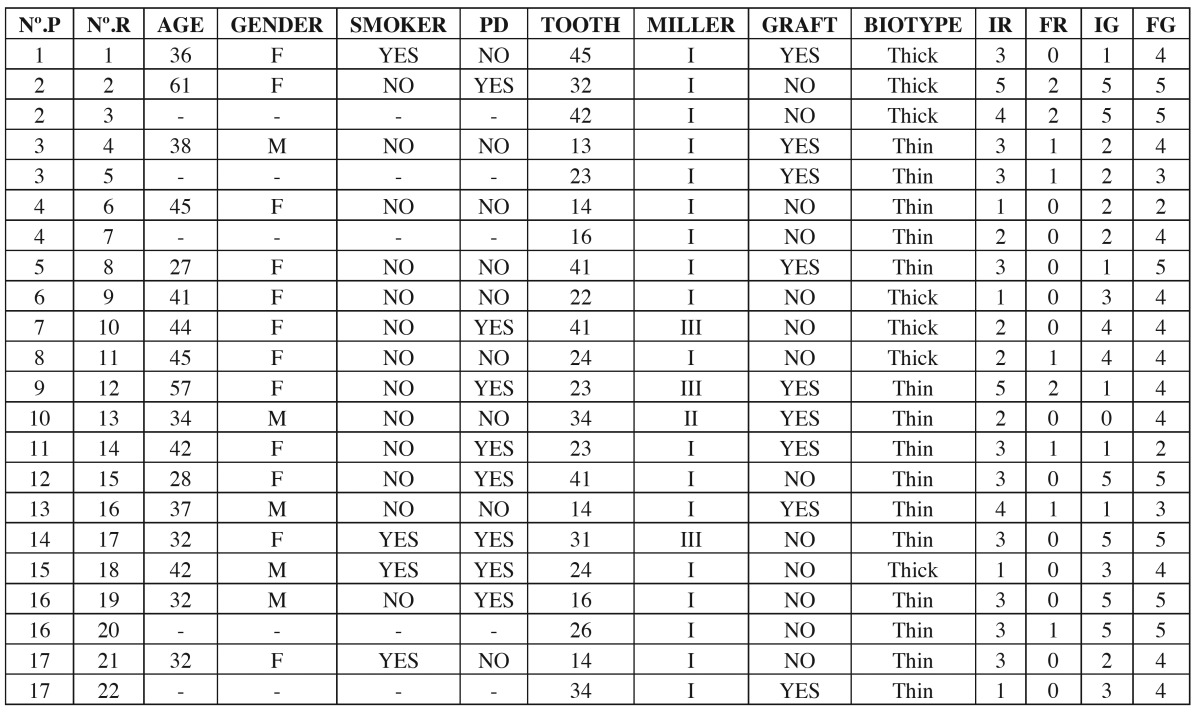
Patient clinical data. NºP. Number of patients evaluated. NºR. Number of recessions evaluated. PD. Periodontal disease. MILLER. Type of gingival recession (I, II or III) according to the Miller classification (16). IR. Initial recession height. FR. Final recession height. IG. Initial keratinized gingiva height apical to the recession. FG. Final keratinized gingiva height apical to the recession.

The following clinical parameters were evaluated:

Preoperative.

- Miller recession class (I, II or III) (16):

Class I: Marginal tissue loss not extending beyond the mucogingival line. No loss of bone or soft tissues in the interdental spaces.

Class II: Marginal tissue loss extending beyond the mucogingival line. No loss of bone or soft tissues in the interdental spaces.

Class III: Marginal tissue loss extending beyond the mucogingival line. Loss of support (bone or soft tissue) in the interdental spaces or malpositioned teeth. The loss of interproximal bone or soft tissue is apical to the cementoenamel junction (CEJ) but coronal to the apical extension of the recession.

- Initial recession (IR): Measured in mm from the CEJ to the most apical gingival margin of the recession.

- Initial keratinized gingiva (IG): Measured in mm from the most apical gingival margin of the recession to the mucogingival line (ML).

Intraoperative:

- Periodontal biotype: Classified as thick (≥ 2 mm) or thin (< 2 mm) at surgery.

Eighteen months after surgery.

- Final recession (FR): Measured in mm from the CEJ to the most apical gingival margin of the recession.

- Final keratinized gingiva (FG): Measured in mm from the most apical gingival margin of the recession to the mucogingival line.

The parameters were all measured using a North Carolina periodontal probe, and the data were obtained from the surgical reports prepared by the examiner (R.C.), the patient case histories and the respective control visits.

- Primary and secondary endpoints

The following primary endpoints were established: gingival recession height and height of the keratinized gingiva apical to the gingival recession.

Both parameters were measured (in mm) before and after surgery, and the observed variation was regarded as the most representative outcome of the different surgical treatments in relation to gingival recession. The following differences (in mm) were calculated: Initial recession (IR) - Final recession (FR) = Recession reduction (RR); and Final gingiva (FG) - Initial gingiva (IG) = Gingival gain (GG). In turn, a third primary endpoint was defined for the statistical analysis: Total recession reduction (TRR)(when the final recession height = 0).

The following secondary endpoints were established: Patient age, gender, smoking habit, periodontal disease, biotype, and Miller recession class, in order to assess their effects upon the primary endpoints and determine the homogeneity of the groups.

- Surgical procedure

**Figure 1:**
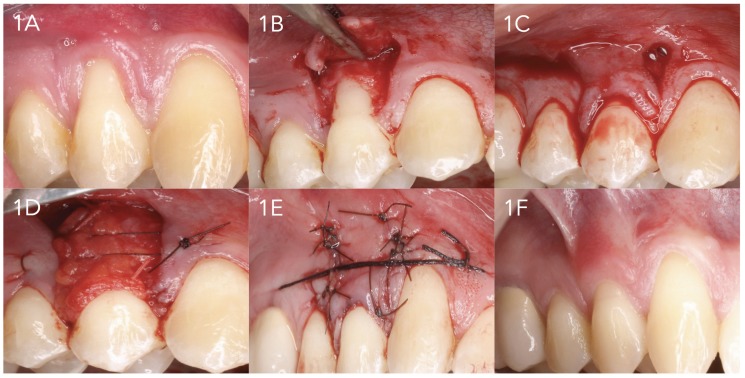
1A. Initial recession. 1B. Raising of the partial thickness flap. 1C. Flap positioned without tension at cementoenamel junction level. 1D. Suturing of the palatal connective tissue graft in the receptor bed. 1E. Flap suturing. 1F. Final condition after 18 months.

**Figure 2:**
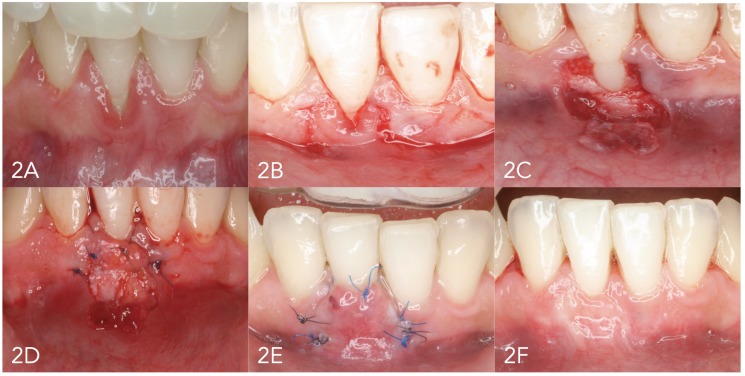
2A. Initial recession. 2B. Flap design. 2C. Raising of the partial thickness flap. 2D. Suturing of the connective tissue graft in the receptor bed. 2E. Flap suturing. 2F. Final condition after 18 months.

A 15C scalpel was used to prepare the coronally advanced flap as described by De Sanctis and Zucchelli in 2007 (17) (Figs. 1A-D and 2A-D). Vertical releasing incisions were made, followed by raising of the partial thickness flap. The muscle plane was freed in order to position the flap coronally without tension, and the exposed root surface was smoothed and decontaminated with root smoothing drills.

In TG the graft covered the root, extending at least 2 mm over the exposed periosteal bed, and was positioned at CEJ level in all cases. The graft was always 1 mm in thickness.

The graft was adapted and sutured using reabsorbable 5/0 suture with dental crown suspending sutures or using simple suturing to the de-epithelialized papillae and simple or mattress sutures internal to the surrounding periosteum.

The flap was positioned coronally at CEJ level and sutured with 5/0 monofilament suture using dental crown suspending sutures and simple suturing to close the releasing incisions.

Tooth brushing of the operated zone was suspended for 15 days in order to avoid possible trauma, and 0.2% chlorhexidine digluconate rinses were prescribed during 15 days, with amoxicillin 500 mg (1 tablet/8 h for 7 days) and ibuprofen 600 mg (1 tablet/8 h for 3 days). The sutures were removed after 15 days, with controls once a week during the first month.

- Statistical analysis

A descriptive analysis was made of the study variables, based on the usual statistics (mean, standard deviation, range and median in the case of quantitative variables, and absolute and relative frequencies in the case of categorical variables).

The Wilcoxon test for related samples was used in application to each surgical technique to assess changes in the recession and gingival values, i.e., to explore the efficacy of the surgical treatment provided.

The Mann-Whitney U-test in turn was used to determine whether the distribution of the response values (and their variations) was homogeneous between the two study groups, i.e., to assess differences in the effect of the operation according to the surgical technique used.

Lastly, an analysis was also made of the influence of other secondary variables referred to the patient profile, habits and clinical conditions upon the effects of the surgical treatment, with a view to identifying possible significant differences between the techniques.

The two surgical techniques were seen to be applied indistinctly to patients with different profiles, habits and disease severity (biotype and Miller recession class). In order to isolate the possible effect of the surgical technique from other confounding factors, we evaluated the homogeneity of the groups (CG and TG) in relation to age, gender, smoking habit, periodontal disease, biotype and Miller recession class. The Fisher exact test and Mann-Whitney U-test revealed no significant differences in relation to these parameters, i.e., the groups were considered to be homogeneous - though the limited power of the study could influence this conclusion.

## Results

Seventeen patients (12 females and 5 males) with a total of 22 localized gingival recessions were selected. The mean patient age was 39.6 ± 9.3 years (range 27-61). There were four smokers and 13 non-smokers. In turn, 8 patients had controlled periodontal disease and 9 had no periodontal disease. Eleven patients had a thin periodontal biotype while 6 had a thick biotype. The control (CG) and test groups (TG) involved 13 and 9 gingival recessions, respectively.

- Analysis of the reduction of gingival recession

The mean gingival recession in CG was 2.5 ± 1.2 mm initially (i.e., at baseline) versus 0.5 ± 0.7 mm after surgery, representing a mean reduction of 2.1 ± 0.9 mm. The corresponding mean percentage decrease was 86.7 ± 21.2% (*p*=0.001).

The mean gingival recession in TG was 3.0 ± 1.1 mm initially versus 0.7 ± 0.7 mm after surgery, representing a mean reduction of 2.3 ± 0.7 mm. The corresponding mean percentage decrease was 81.7 ± 17.8% (*p*=0.007).

Both techniques were seen to result in significant reduction of gingival recession (Wilcoxon test), though without significant differences between the two surgical procedures ( Table 2).

**Table 2:**
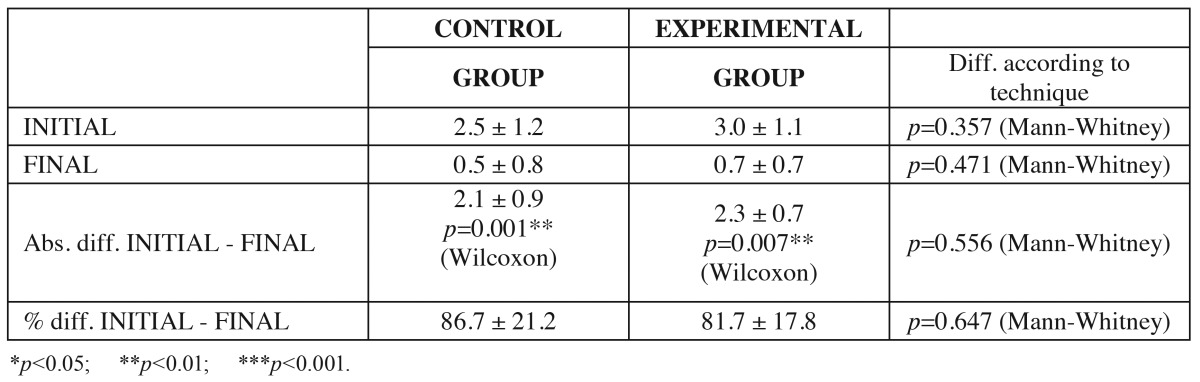
Evaluation of the reduction of gingival recession in the two groups. Wilcoxon test results for initial versus final differences, and Mann-Whitney U-test for differences between techniques.

- Analysis of the gain in keratinized gingiva

The mean keratinized gingival height in CG was 3.8 ± 1.3 mm initially versus 4.3 ± 0.9 mm after surgery, representing a mean gain of 0.5 ± 0.8 mm. The corresponding mean percentage increase was 20.5 ± 37.4% (*p*=0.063).

The mean keratinized gingival height in TG was 1.3 ± 0.9 mm initially versus 3.7 ± 0.9 mm after surgery, representing a mean gain of 2.3 ± 1.2 mm. The corresponding mean percentage increase was 185.4 ± 135.5% (*p*=0.007).

A significant increase in keratinized gingiva was recorded in TG (*p*=0.007; Wilcoxon test). In contrast, no statistically significant variation was noted in CG, though there was a strong tendency towards treatment success (*p*=0.063). The mean gain in keratinized gingiva with grafting was found to be greater than without grafting, in both mm and percentage terms ( Table 3).

**Table 3:**
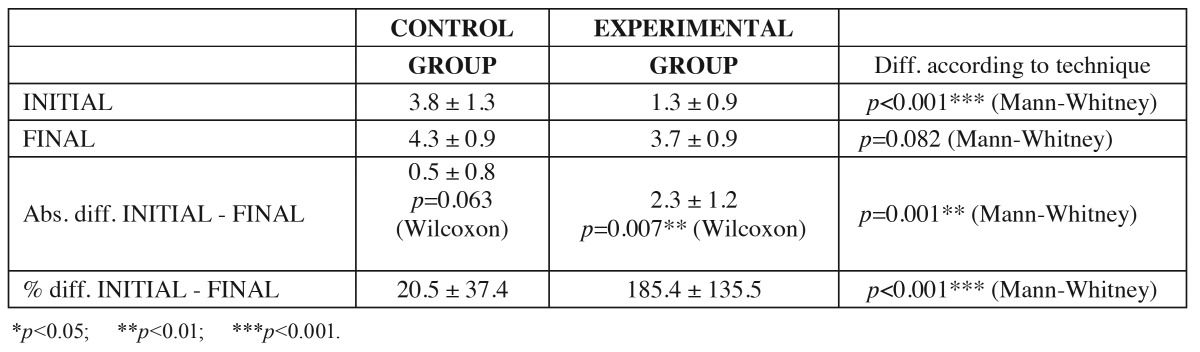
Evaluation of the gain in keratinized gingiva in the two groups. Wilcoxon test results for initial versus final differences, and Mann-Whitney U-test for differences between techniques.

- Analysis of the total reduction of gingival recession

A total reduction of gingival recession (final recession height = 0 mm) was achieved in 13 of the 22 gingival recessions subjected to surgery. The total reduction rate in CG and TG was 69.2% and 44.4%, respectively - the difference between the two groups being non significant (*p*=0.386; Fisher exact test), i.e., there were no differences in terms of total reduction of gingival recession between CG and TG ( Table 4).

**Table 4:**
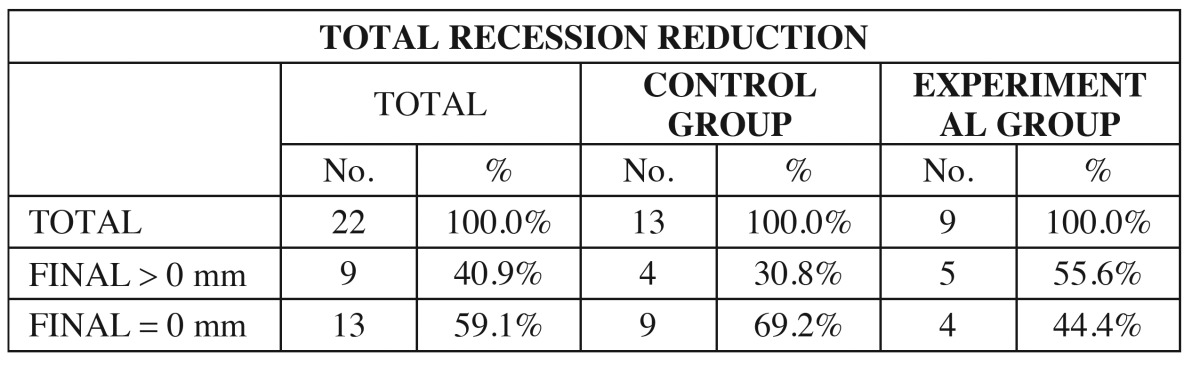
Evaluation of the total reduction of gingival recession in the two groups.

## Discussion

The surgical treatment of gingival recession always should be based on the use of techniques affording scientific evidence of a high percentage of root coverage, with a view to offering patients the best possible outcome. Consensus has recently been reached on the need for at least 5 years of follow-up in order to adequately assess the stability of the clinical results obtained (18).

The literature describes positive results with the surgical treatment of localized gingival recessions using coronally advanced flaps (CAF) associated to subepithelial connective tissue graft (SCTG) (15). However, a number of studies have also shown that there are no significant differences after 6 months in terms of the reduction of gingival recession between CAF alone and CAF plus SCTG, in patients with local recessions (19,20). Despite its limitations, the present study corroborates these results after 18 months of follow-up.

In coincidence with our own observations, Wennstrom and Zucchelli, in a clinical trial involving two years of follow-up, concluded that there are no differences in root coverage between the use of coronally advanced flaps with or without graft (21).

We recorded no significant differences in the reduction of gingival recession between CG and TG, though it must be taken into account that since this is a retrospective study, the patients were not randomly treated with one surgical technique or the other. In effect, the patients either did or did not undergo grafting depending on the severity of the condition (as evidenced by initial gingival recession height and initial keratinized gingiva). This implies that patients with a greater initial recession height or with less keratinized gingiva apical to the recession underwent grafting in addition to coronally advanced flap surgery, while those patients with lesser initial recession height or with more keratinized gingiva apical to the recession did not undergo graft- i.e., as a rule, the more serious gingival recessions were treated by combining the coronally advanced flap technique with graft.

More recently, a systematic review of periodontal plastic surgery came to the same conclusions: the use of a connective tissue graft combined with a coronally advanced flap does not result in better outcomes in terms of root coverage than when only a coronally advanced flap is used (22). However, over the years, slight coronal displacement of the gingival margin is observed in those cases where SCTG was performed, and likewise slight apical contraction is noted in those cases where only CAF was used. This tendency could be related to the keratinized gingival thickness achieved with one technique or the other through modification of the gingival biotype (15). Gingival recessions treated with CAF tend to show increases in terms of root exposure. This may be explained by the tendency of the soft tissues to experience slight contraction during the initial healing phase (23). The presence of a graft beneath the CAF is associated to lesser soft tissue contraction and therefore to an increased probability of achieving total root coverage (20). It has been shown that the decrease in gingival recession achieved with either surgical technique can be maintained, provided adequate maintenance is carried out over a long period of time (24).

In 2007, De Sanctis and Zucchelli published a study in which they evaluated their flap design in application to the treatment of localized gingival recessions (17). The mean root coverage rate was 98.6% in a total of 40 gingival recessions treated with coronally advanced flaps in the absence of graft, and total reduction of recession was achieved in 88% of the cases. In our series, the patients in which grafting was not performed showed a mean root coverage rate of 86.7%, and total reduction of recession was achieved in 69.2% of the cases. On the other hand, the results of the above mentioned study are in contrast to our own findings as regards the gain in gingival attachment in cases without graft. In this regard, De Sanctis and Zucchelli recorded an inverse relationship between the initial and final keratinized gingival height. In our study, surgery without connective tissue graft was associated to similar keratinized gingiva values before and after the operation. In any case, regarding the increase in keratinized gingiva, and on comparing CAF versus CAF combined with SCTG, Cortellini *et al*. (2009) describes an important difference in favor of surgery associated to graft (20), in coincidence with our own findings.

It is also important to take into account that the patients in TG presented more severe initial gingival recession (*p*<0.001; Mann-Whitney U-test). This can be taken to imply that the use of connective tissue graft results in a greater increase in keratinized gingiva even with initial conditions characterized by lesser initial keratinized gingival heights compared with CG.

On the other hand, as regards the total reduction of gingival recession, our study showed no significant differences between CAF with or without connective tissue graft. However, other studies such as those published by Da Silva *et al*. (2004) and Cortellini *et al*. (2009), and the systematic review conducted by Cairo (2008), have reported greater success rates in terms of the total reduction of localized gingival recession when using CAF combined with SCTG (19,20,15). Nevertheless, other clinical trials comparing the two techniques and involving 5 years of follow-up, but centered on generalized gingival recession, likewise have found no differences in terms of the total reduction of gingival recession (25).

## Conclusion

In our study, both surgical techniques were found to be effective in application to localized gingival recessions, with no significant differences in root coverage after 18 months between the use of a coronally advanced flap alone versus a coronally advanced flap combined with subepithelial connective tissue graft. The use of such connective tissue grafts did not improve upon the reduction of gingival recession, but was associated to an increased gain in keratinized gingiva.
